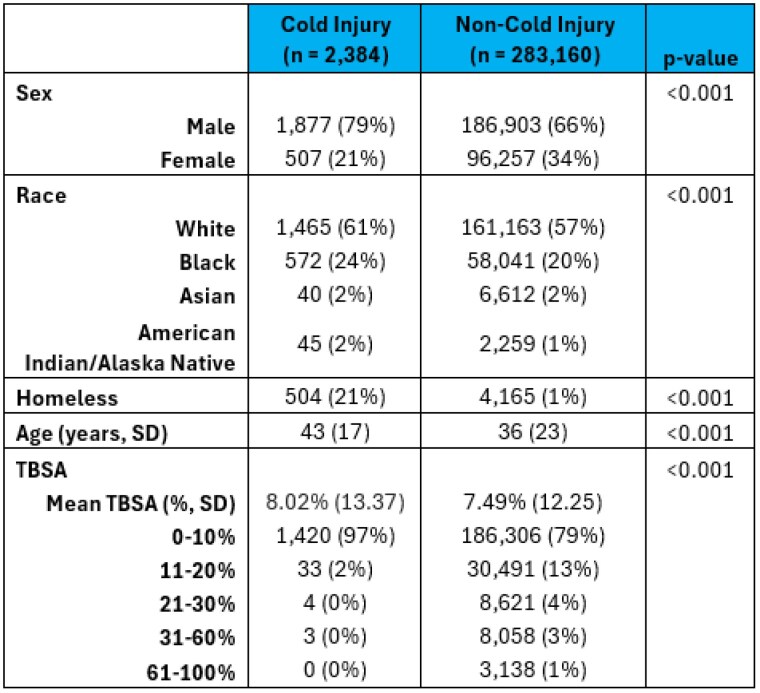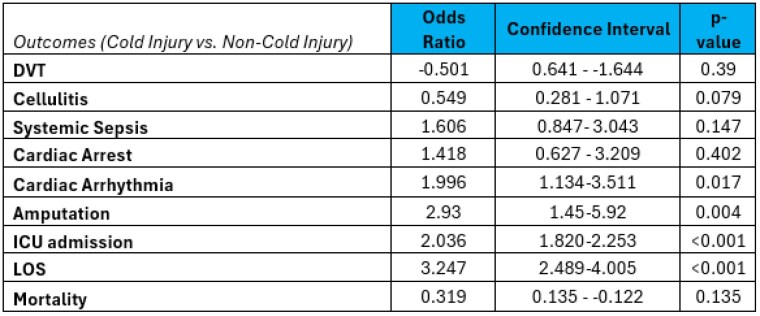# 561 Analysis of Cold Injury Admissions and Their Complications Across North America

**DOI:** 10.1093/jbcr/iraf019.190

**Published:** 2025-04-01

**Authors:** Tyler Murphy, Arman Fijany, Emily Swafford, Jordan Garcia, Punit Vyas, Robel Beyene, Stephen Gondek, Anne Wagner, Elizabeth Slater

**Affiliations:** Vanderbilt Burn Center; Vanderbilt University Medical Center; Vanderbilt Burn Center; Vanderbilt University Medical Center; Vanderbilt University Medical Center; Vanderbilt University Medical Center; Vanderbilt University Medical Center; Vanderbilt University Medical Center; Vanderbilt University Medical Center

## Abstract

**Introduction:**

Cold injuries are amongst the most challenging injury types to manage. These injuries are unique in presentation, initial assessment, and treatment algorithms due to locations of exposure on the body and how cold effects systemic perfusion. In this nationwide database study, we hypothesize that cold injury patients will have increased rates of prolonged admissions and infectious complications as compared to their burn counterparts.

**Methods:**

The American Burn Association (ABA) Noncommercial Burn Research Dataset (NBR) was investigated for burn center admissions due to cold injuries from 2012-2021. Primary outcomes included ICU admission and hospital length of stay (LOS). Secondary outcomes included amputation, deep venous thrombosis (DVT), cellulitis, sepsis, cardiac arrhythmia, and cardiac arrest. All binary outcomes were analyzed with logistic multivariate regression, apart from hospital length of stay, which was modeled with linear regression. Covariates for regression analysis included age, sex, inhalation injury, and percent total body surface area (TBSA) of injury.

**Results:**

A total of 285,044 patients were identified within the NBR. Of these, 2,384 patients experienced cold injury. These patients were statistically more likely to be older (mean 43 years [SD 17] vs. 36 years [23]) homeless (21% vs. 1%) males (79% vs. 21%) with a larger mean TBSA (8.0% [13] vs. 7.5% [12]). Patients were significantly more likely to need an ICU admission (odds ratio [OR 2.0]; 95% confidence interval [CI] 1.820,2.253) with a longer length of stay by 3 days (OR 3.2; CI 2.489,4.005). In multivariate analysis, cold injury patients had a greater likelihood of cardiac arrhythmia (OR 2.0; CI 1.13,3.51) and amputation (OR 2.9; 1.45,5.92). There were no significant differences in mortality.

**Conclusions:**

Cold injuries are associated with significantly extended LOS and higher rates of cardiac arrhythmia and amputation. The extended LOS is likely due to the predisposition of homelessness in the cold injury population and short-term disability following the increase in amputation. The associated increase in cardiac arrhythmia warrants further investigation to determine its association with comorbid hypothermia or if there is a systemic effect of extremity cold injury not previously documented in the literature.

**Applicability of Research to Practice:**

Our data helps burn physicians determine the trajectory of cold injured patients in reference to complications and LOS in a nationwide database study. The novel association between cold injury and cardiac arrhythmia should be noted and further investigated to improve outcomes of patients.

**Funding for the Study:**

N/A